# Construction and Characterization of Adenovirus Vectors Encoding Aspartate-*β*-Hydroxylase to Preliminary Application in Immunotherapy of Hepatocellular Carcinoma

**DOI:** 10.1155/2018/9832467

**Published:** 2018-07-15

**Authors:** Yujiao Zhou, Feifei Liu, Chengmin Li, Guo Shi, Xiaolei Xu, Xue Luo, Yuanling Zhang, Jingjie Fu, Aizhong Zeng, Limin Chen

**Affiliations:** ^1^Department of Infectious Disease, The First Affiliated Hospital of Chongqing Medical University, Chongqing 400016, China; ^2^Department of Infectious Disease, The People's Hospital of Deyang City, Deyang, Sichuan Province 618000, China; ^3^Department of Infectious Disease, The Chongqing Fuling Center Hospital, Chongqing 400016, China; ^4^Department of General Surgery, The First Affiliated Hospital of Chongqing Medical University, Chongqing 400016, China; ^5^Toronto General Research Institute, University of Toronto, Toronto, ON, Canada

## Abstract

Dendritic cells (DCs) harboring tumor-associated antigen are supposed to be a potential immunotherapy for hepatocellular carcinoma (HCC). Aspartate-*β*-hydroxylase (AAH), an overexpressed tumor-associated cell surface protein, is considered as a promising biomarker and therapeutic target for HCC. In this study, we constructed adenovirus vector encoding AAH gene by gateway recombinant cloning technology and preliminarily explored the antitumor effects of DC vaccines harboring AAH. Firstly, the total AAH mRNA was extracted from human HCC tissues; the cDNA was amplified by RT-PCR, verified, and sequenced after TA cloning. Gateway technology was used and the obtained 18T-AAH was used as a substrate, to yield the final expression vector Ad-AAH-IRES2-EGFP. Secondly, bone marrow-derived DCs were infected by Ad-AAH-IRES2-EGFP to yield AAH-DC vaccines. Matured DCs were demonstrated by increased expression of CD11c, CD80, and MHC-II costimulatory molecules. A dramatically cell-killing effect of T lymphocytes coculturing with AAH-DCs on HepG2 HCC cell line was demonstrated by CCK-8 and FCM assays in vitro. More importantly, in an animal experiment, the lysis effect of cytotoxic T lymphocytes (CTLs) on HepG2 cells in the AAH-DC group was stronger than that in the control groups. In conclusion, the gateway recombinant cloning technology is a powerful method of constructing adenovirus vector, and the product Ad-AAH-IRES2-EGFP may present as a potential candidate for DC-based immunotherapy of HCC.

## 1. Introduction

Hepatocellular carcinoma (HCC) is a common malignant tumor, and despite curable strategies such as resection or liver transplantation, patients with advanced HCC continue to present a poor outcome [1, 2]. Although sorafenib has been shown to improve survival of advanced HCC patients, it ultimately failed to show any improvement in outcomes for the treatment of HCC in randomized studies. The clinical trials of new drugs including lenvatinib, regorafenib, and pembrolizumab have shown promise; however, more convictive clinical evidences are still needed [3]. Therefore, there is a clear need for new therapeutic approaches for HCC.

Aspartate-*β*-hydroxylase (AAH) is a highly conserved type II transmembrane protein and a kind of *α*-ketoglutarate-dependent dioxygenase. Currently, AAH has been found highly expressed in a variety of human malignancies including HCC, cholangiocellular carcinoma, and breast, pancreatic, and non-small lung cancer [4–7]; however, it is rarely expressed in normal tissues and lowly expressed in tumor-adjacent tissues [4]. Previous researches have demonstrated that overexpression of AAH strikingly increases motility and invasiveness of HCC cell lines [8]. Xian et al. [9] reported that AAH overexpression was detected in 150 of 161 patients with HCC, and higher expression levels of AAH correlated significantly with the presence of intrahepatic metastasis and the progression of histological grades. Another clinical study showed that overexpression of AAH is associated with worse clinical outcomes in HCC patients after surgery [10]. The research of Aihara et al. [11] has demonstrated that small molecule inhibitors of AAH produce antitumor effect in HCC. As a conclusion, AAH could be considered as a prognostic maker and therapeutic target for HCC.

After forty years of research, it is generally realized that dendritic cells (DCs), the most powerful professional antigen-presenting cells, are the center part of the immune system, because of their ability to control both immune tolerance and immunity. The application of DCs loaded with tumor-associated antigen (TAA) is supposed to be a promising approach in the prophylaxis and therapy of malignant tumors. The prophylactic and therapeutic effect of DC-mediated tumor vaccines has been successfully confirmed in mouse models. Besides, the history of application of therapeutic DC vaccines in cancer patients has been more than a decade. To date, several new-type DC vaccines have successfully gained entry to phase III clinical trials; however, the most appropriate indication for these vaccines is melanoma [12]. Delivering TAA into DCs in vitro needs the help of vectors. Diverse tools including cytoplasmic transduction peptide delivery system, microbial components, plasmids, and virus have been developed [13–16]. In this study, our aim was to construct a novel adenovirus (Ad) vector encoding AAH gene and to preliminarily explore the potential antitumor activity of DCs loaded with AAH against HCC.

## 2. Materials and Methods

### 2.1. Primer Designing and Polymerase Chain Reaction (PCR) Amplification of DNA Fragments

Primers (Table 1) were synthesized according to the human AAH gene sequence (GenBank accession number 004318) and obtained commercially from the manufacturer (Invitrogen, USA, HG1606160040). Total mRNA was extracted from human HCC tissues (supplied by the Hepatobiliary Surgery Department of the First Affiliated Hospital of Chongqing Medical University). PCR amplification system included 5x PrimeSTAR® GXL Buffer 10 *μ*l, dNTP mixture 1 *μ*l, forward primer 0.5 *μ*l, reverse primer 0.5 *μ*l, PrimeSTAR GXL for RT-PCR 2 *μ*l, DNA 2 *μ*l, and RNase-free dH_2_O 34 *μ*l. PCR amplification conditions were as follows: 98°C for 10s; 68°C for 15 s; 68°C for 2 min, 30 cycles; and 4°C for 3 min. Subsequently, PCR products were cloned into pMD18-T vector using a TA cloning kit (Takara, Japan, R023A) and sequenced. The plasmid 18T-AAH obtained by TA cloning would be used as templates for the following PCR amplification.

### 2.2. Construction of Ad Vectors by Gateway Technology

**Figure 1:**
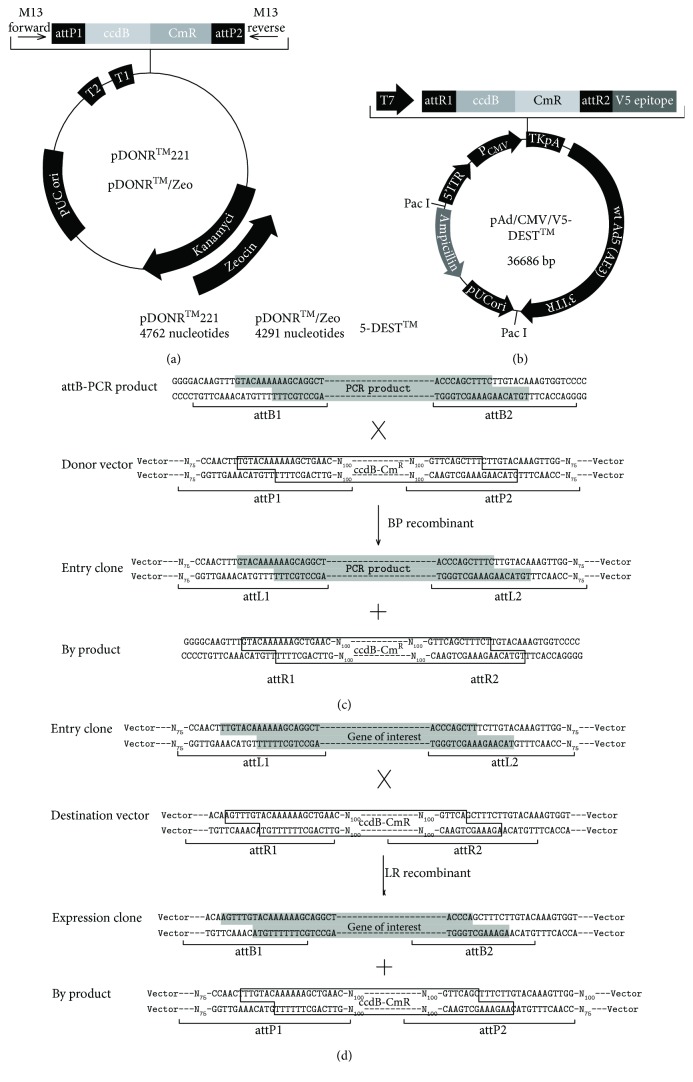
Images of vectors and brief flow chart of gateway cloning method. (a) Donor vector pDONR221. (b) Destination vector pAd CMV/V5-DEST. (c) The BP recombinant (attB × attP → attL × attR). (d) The LR recombinant (attL × attR → attB × attP).

Firstly, 18T-AAH and plasmid internal ribozyme entry site-2/enhanced green fluorescent protein (pIRES2-EGFP) were used as templates, respectively; the products attB1-AAH and attB2-IRES2-EGFP were amplified by PCR. Secondly, the objective DNA fragments flanked by attB sites were obtained by PCR amplification using the primers shown in Table 2. Finally, recombinant Ad vector encoding AAH was produced by gateway technology according to the manufacturer's instruction (Takara, Japan, R023A). Briefly, in the BP reaction, PCR products before-mentioned were inserted into the donor vector with the attP sites (Figure 1(a)) yielding the shuttle vector, containing the gene of interest flanked by the attL sites. Then, in the LR reaction, the shuttle vector was integrated into the destination vector (pAd CMV/V5-DEST) (Figure 1(b)) containing the attR sites producing the final expression constructs (Ad-AAH-IRES2-EGFP). The brief flow chart was shown in Figures 1(c) and 1(d). The final products were also transformed into competent *E. coli* DH5*α* and positively selected by DNA sequencing.

### 2.3. Ad Packaging and Amplification

The recombinant plasmid Ad-AAH-IRES2-EGFP was linearized by PacI and delivered into HEK293 cells for Ad packaging and amplification. HEK293 cells were seeded at 4.5 × 10^5^ cells per well into a 6-well plate with DMEM media containing 10% fetal bovine serum (FBS) (Gibco, USA, 10099141) and 1% penicillin streptomycin at 37°C in a humidified atmosphere of 5% CO_2_. The infection process was accomplished according to the instruction of Lipofectamine 2000 transfection handbook (Invitrogen, USA). Postinfected HEK293 cells represented a significant cytopathic effect (CPE) at day 8 and were cracked by repeated freezing and thawing between 37°C and −80°C for three times aiming to release the viral particles. The supernatants containing virus was collected and stored at −80°C. The titer of Ad-AAH-IRES2-EGFP was detected by immunoassay. In brief, HEK293 cells were seeded into a 24-well plate at a concentration of 3 × 10^5^ cells/well. Subsequently, the virus was diluted at a dilution range from 10^−2^ to 10^−6^ by DMEM medium and infected HEK293 cells, respectively. After 48 h incubation, AAH polyclonal antibody (1 : 1000, Proteintech, USA, 271391) was added into each well. The infected cells represented an obvious brown color after 3,3′-diaminobenzidine solution dyeing, then the titer was calculated by the formula: (mean positive cells/per field) × (fields/per well)/(volume of virus × dilution ratio).

### 2.4. Construction of AAH-DC Vaccine

In brief, the bone marrow was flushed from the femurs, and the tibias of C57BL/6 mice were depleted of erythrocytes with lysis buffer. The cells obtained were cultured in a 6-well plate with RPMI-1640 media containing 10%FBS, 10 ng/ml recombinant murine GM-CSF (PeproTech, USA, 081455), and 10 ng/ml recombinant murine IL-4 (PeproTech, USA, 081449). Half of the media were replaced with fresh cytokine-containing (rmGM-CSF and rmIL-4) media at a one-day interval. On days 5 and 6, 10 ng/ml recombinant murine TNF-*α* (PeproTech, USA, 061454) was added to the media. On day 7, nonadherent cells were harvested, resuspended in sterile phosphate-buffered saline (PBS), and seeded onto a 12-well plate at a density of 5 × 10^5^ cells/well. Ad-AAH-IRES2-EGFP was added to the well at a multiplicity of infection (MOI) of 200. After 48 hours of incubation, AAH-loaded DCs were obtained. DCs incubated with empty vector Ad-IRES2-EGFP (supplied by the manufacturer) or alone served as the control.

The phenotype analysis of maturated DCs was identified by flow cytometry (FCM). At 36 h postinfection, DCs were collected and adjusted into a concentration of 1 × 10^6^/ml in 0.2 ml PBS. Cells were cocultured with anti-mouse CD11c-PerCP-Cy5.5 (eBioscience, USA, 450114), CD80-PE (eBioscience, USA, 120801), and MHC-II-APC antibodies (eBioscience, USA, 175321) at 4°C for 30 min. The cells were then washed twice with PBS buffer; the expression of mature markers of DCs was analyzed using FCM.

### 2.5. Cell-Killing Effect In Vitro

At first, splenic T cells derived from C57BL/6 mice were cultured in a 6-well plate at 1 × 10^6^ cells/well with complete RPMI-1640 media, then cocultured with AAH-DC vaccines, GFP-DC vaccines, and blank DC vaccines at a ratio of 1 : 20, respectively. The three kinds of mix-cultured cells were severally harvested after 72 hours of incubation and used as the effector cells.

The human HCC cell line HepG2, highly expressing AAH, was maintained in DMEM medium supplemented with 10% FBS. The cellular proliferation activity was detected by cell counting kit-8 (CCK-8) (Genview, USA, GK3607) assay. Briefly, HepG2 cells were used as the target cells, cultured in a 96-well plate at 3 × 10^3^ cells per well, and mixed with effector cells obtained from each of groups abovementioned at effector-to-target (E/T) ratios of 40 : 1, 20 : 1, 10 : 1, or 5 : 1. After 24 hours of cocultivation, the supernatants were removed and the adherent cells were gently washed once by sterile PBS buffer. The CCK-8 solution was added to all the wells according to the directions of the manufacturer. The OD value was detected at 450 nm by applying a microplate reader. HepG2 cells alone were used as the blank control group.

On the other hand, HepG2 cells were seeded in a 6-well plate at 1 × 10^5^ cells/well, cocultured with different kinds of effector cells aforementioned at a ratio of 40 : 1. HepG2 cells alone were also used as control. After 24 hours of incubation, the supernatants were removed. The four groups of target cells were harvested, dissociated with 0.25% trypsin, and resuspended in Annexin V-FITC/PI binding buffer. The cellular apoptosis rate of target cells was detected by FCM.

### 2.6. Lactate Dehydrogenase (LDH) Release Assay

6~8-week-old female C57BL/6 mice, purchased from the Animal Experimental Center of Chongqing Medical University, were maintained under individual ventilated cage (IVC) conditions, fed standard chow, and supplied with sterilized water. All the procedures were approved by the Ethics Committee of the First Affiliated Hospital of Chongqing Medical University. All the mice were in deep anesthesia by diethyl ether and euthanized by high concentration of CO_2_ inhalation. C57BL/6 mice were randomly divided into five groups (3 mice/group): (1) AAH-DC group, the mice were immunized by subcutaneous injection with 1 × 10^6^ AAH-DCs in 0.1 ml PBS; (2) GFP-DC group, the mice were immunized by subcutaneous injection with 1 × 10^6^ GFP-DCs in 0.1 ml PBS; (3) DC group, the mice were subcutaneously injected with 1 × 10^6^ noninfected DCs in 0.1 ml PBS; (4) PBS group, the mice were subcutaneously injected with 0.1 ml sterile PBS; and (5) AAH group, Ad-AAH-IRES2-EGFP alone in a volume of 0.1 ml was used to perform the immunization. The injection was given three times at a 7-day interval. The splenic T cells derived from C57BL/6 mice of each group were, respectively, acquired after the last immunization.

Cytotoxic function of the T cells was determined according to LDH Cytotoxicity Assay Kit (Beyotime, China, 092017170925). All steps were performed following the manufacturer's instructions. Briefly, the activated T lymphocytes, obtained after coculturing with 25 *μ*g/ml mitomycin-C-treated HepG2 cells and 5 ng/ml recombinant murine IL-2 (Peprotech, USA, 0608108) for 72 hours, were regarded as the effector cells. HepG2 cells were used as target cells, seeded on a 96-well plate at a density of 4 × 10^3^ cells/well, and cocultured with effector cells at the E/T ratios of 50 : 1, 100 : 1, 200 : 1, and 400 : 1 in a total volume of 200 *μ*l/well for 24 hours of incubation. The spontaneous release of LDH by target cells or effector cells was assayed by incubation of target cells or effector cells alone, respectively. The maximum release of LDH was determined by mixing the target cells with lysis solution. The supernatants were measured by LDH working solution, and absorbance was detected at 490 nm using a microplate reader. The cellular lysis rate was calculated as the following formula: cytotoxicity (%) = [OD (experimental) − OD (effector spontaneous) − OD (target spontaneous)]/[OD (target maximum) − OD (target spontaneous)] × 100.

### 2.7. Statistical Analysis

The results were expressed as means ± SD and analyzed with SPSS 21.0 statistical software package (SPSS Inc., Chicago, IL, USA). Data was analyzed by one-way analysis of variance (ANOVA) followed by a LSD *t*-test when comparing more than two groups. *P* < 0.05 was considered significant.

## 3. Results

### 3.1. Amplification of AAH Gene by PCR

**Figure 2:**
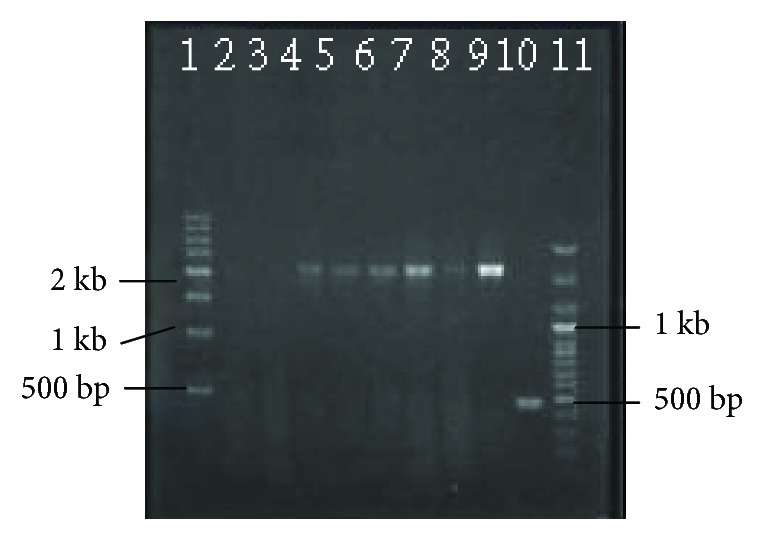
Agarose gel electrophoresis of gradient PCR products. The total AAH mRNA was extracted from surgically resected HCC tissues; the PCR products were amplified with the use of commercially obtained primers and validated by 0.8% agarose gel electrophoresis. Lane 1: 500 bp marker; lane 11: 100 bp marker; lanes 3–9: gradient AAH PCR products, respectively, representing annealing temperature: 56°C, 58°C, 60°C, 62°C, 64°C, 66°C, 68°C; lane 2: negative control group; lane 10: positive control group, 462 bp.

The DNA fragment of the AAH gene was amplified by reverse transcription polymerase chain reaction (RT-PCR). The expected band size was of 2270 bp. According to gradient PCR, 68°C was found as the optimum annealing temperature (Figure 2).

### 3.2. Identification of Ad Vector Encoding AAH

**Figure 3:**
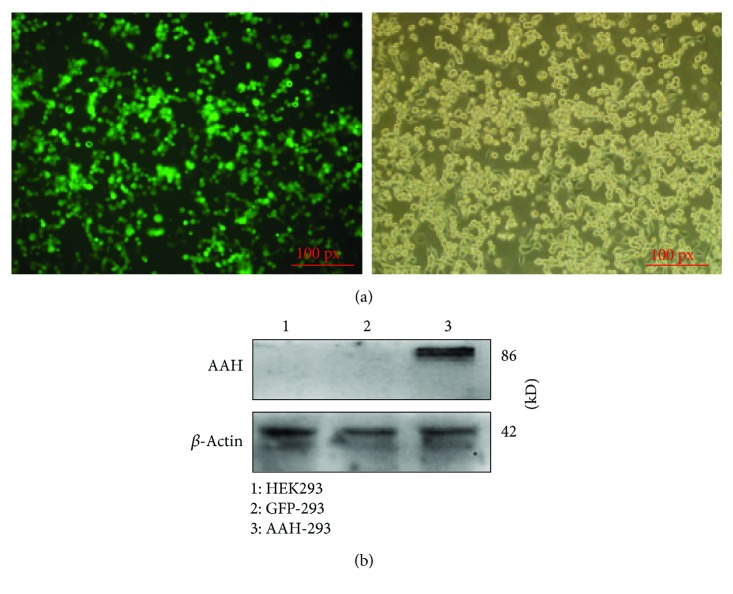
Expression of AAH in HEK293 cells after infection. (a) The fluorescent (①) and visible (②) photograph of HEK293 cells infected by Ad-AAH-IRES2-EGFP at 13 d postinfection (100x). (b) The expression of AAH protein in HEK293 cells at 48 h postinfection by western blot.

The recombinant Ad vector encoding AAH identified by PCR and DNA sequencing was selected and packaged into HEK293 cells. After 13 days of conventional cultivation, infected HEK293 cells showed significant CPE and clear green fluorescence (Figure 3(a)). The titer of Ad-AAH-IRES2-EGFP was detected as 5.0 × 10^9^ ifu/ml according to the immunoassay aforementioned. Western blot results as shown in Figure 3(b) revealed that the positive expression of AAH protein (~86 kD) was detected in HEK293 cells infected by Ad-AAH-IRES2-EGFP; no expression was found in control groups.

### 3.3. Identification of DC Vaccines

**Figure 4:**
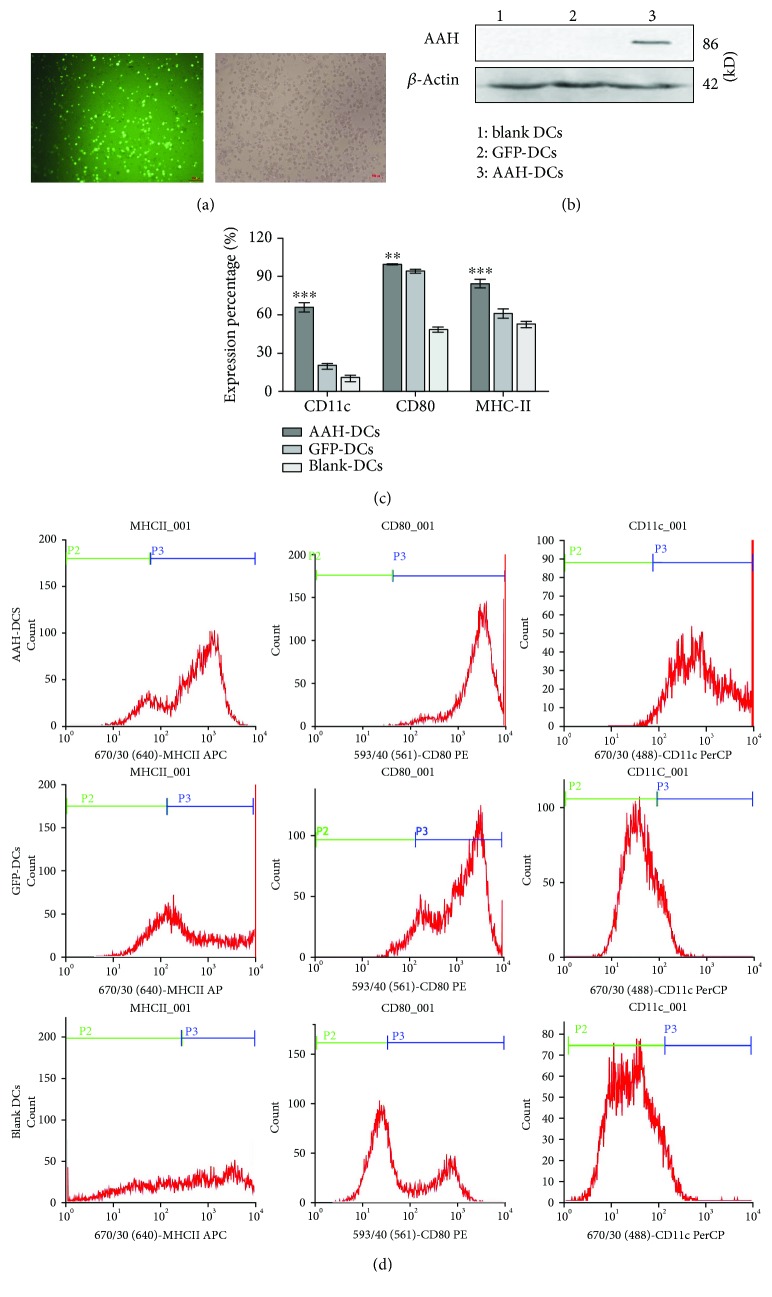
Expression of the AAH gene in dendritic cell (DC) vaccines. (a) The fluorescent (①) and visible (②) images of DCs infected by Ad-AAH-IRES2-EGFP at a MOI of 200 after 36 h infection (100x). (b) Expression of AAH protein in DC vaccines by western blot. (c) The expression percentage of typical phenotypes of mature DCs by FCM analysis. Compared to the GFP-DC group or blank DC group, the maturation markers of DCs in the AAH-DC group were significantly highly expressed; ^∗∗∗^*P* < 0.001, ^∗∗^*P* < 0.01. (d) The typical phenotypes of DCs by FCM.

The expression of green fluorescent protein in DCs was observed by inverted fluorescence microscope (Figure 4(a)), indicating that recombinant Ad vector harboring AAH could effectively infect DCs. The expression of AAH protein in postinfected DCs was also measured by western blot. As shown in Figure 4(b), AAH protein was obviously expressed in the AAH-DC group, while there was no expression in other groups. In addition, compared to GFP-DCs or blank DCs, a significant increased expression of CD11c, CD80, and MHC-II in AAH-DCs was found by FCM analysis (Figures 4(c) and 4(d)).

### 3.4. CTL Activity

**Figure 5:**
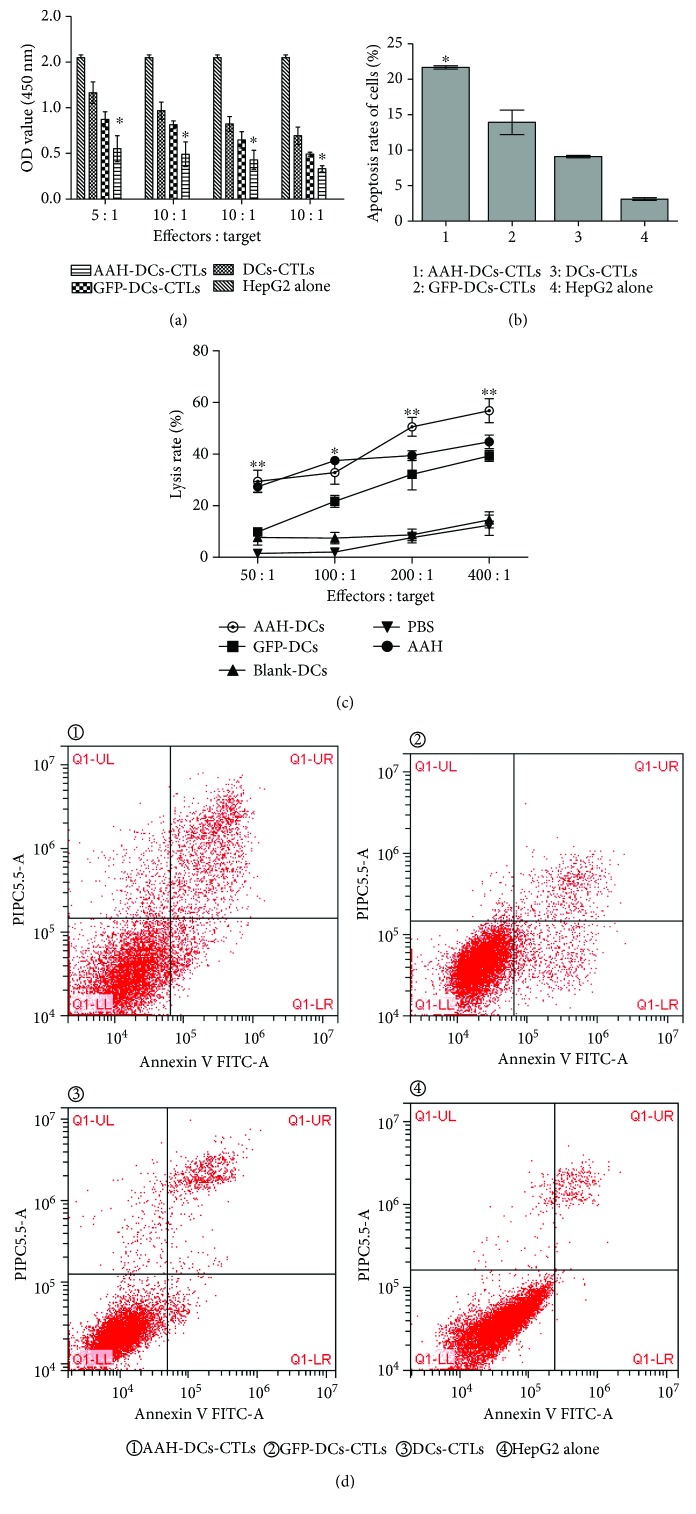
CTL activity. T cells cocultured with AAH-DCs, GFP-DCs, or blank DCs for 72 h were regarded as effector cells, respectively. HepG2 cells were used as target cells. On the other hand, AAH-DCs, GFP-DCs, and blank DCs were applied as vaccines to immunize C57BL/6 mice, respectively. The experiments were performed in triplicate, and the bars represent the mean ± SD. (a) The effect of T cells cocultured with DCs on the proliferation of HepG2 cells by CCK-8 analysis. ^∗^*P* < 0.05, when compared with other groups. (b) The proapoptosis effect of T cells cocultured with DCs on HepG2 cells by FCM. ^∗^*P* < 0.05, when compared with the control groups. (c) The lysis effect of CTLs derived from immunized mice against HepG2 cells. ^∗^*P* < 0.05, ^∗∗^*P* < 0.01 when compared with other groups except the AAH group. According to our results, although there was no statistical differences between the AAH-DCs group and the AAH group, the lysis effect of the AAH-DC vaccine against HepG2 cells was stronger than that of the AAH vaccine. (d) The picture of apoptosis of target cells by FCM.

According to the results of the CCK-8 experiment (Figure 5(a)), the proliferation activity of HepG2 cells was gradually decreasing following with the increasing E/T ratio. At each E/T ratio, the proliferation of HepG2 cells in the AAH-DC-CTL group was lower than that in the control groups. On the other hand, FCM analysis revealed that the apoptosis rate of target cells in the AAH-DC-CTL group (21.68 ± 0.24%) was higher than that in the GFP-DC-CTL group (13.93 ± 1.73%), DC-CTL group (9.09 ± 1.07%), and HepG2 alone group (3.10 ± 1.20%); the differences were statistically significant (*F* = 237.641, *P* = 0.001) (Figure 5(b)). In addition, we prepared an LDH release assay to evaluate the cytotoxic activity of CTLs. The results showed that the lysis rate of target cells in the AAH-DC group at E/T ratios of 50 : 1, 100 : 1, 200 : 1, and 400 : 1 was 29.40 ± 7.46%, 32.83 ± 7.76%, 50.63 ± 6.28%, and 56.80 ± 8.12%, respectively. Meanwhile, the highest lysis rate of the AAH-DC group at an E/T ratio of 400 : 1 was significantly higher than that of the GFP-DC group (39.30 ± 3.72%), DC group (14.53 ± 5.40%), PBS group (12.43 ± 6.87%), and AAH group (44.80 ± 4.45%) (Figure 5(c)). Although the percentage of cytotoxicity in the AAH group was higher than that in the GFP-DC group, DC group, and PBS group, it is still lower than that in the AAH-DC group, indicating that DCs harboring AAH gene were prone to induce a stronger CTL immune response in vivo.

## 4. Discussion

The success of cancer immunotherapy relies on the existence of a high sensitive and high specific TAA. Currently, with respect to HCC, a number of TAAs have been identified: *α*-fetoprotein (AFP), New York esophageal squamous cell carcinoma-1 (NY-ESO-1), melanoma antigen gene (MAGE) family, glypican-3 (GPC3), and so on [17]. T cell responses targeting those TAAs in HCC patients can be generally observed; however, the subsequent clinical outcomes are not very satisfactory [18]. The AAH gene is also well characterized in HCC, which has been described in detail earlier in the article. Noda et al. [19] reported that immunization of rats with AAH-loaded DCs generated cytotoxicity against intrahepatic cholangiocarcinoma produced by hepatic injection of BDEneu-CL24 cells, which was probably associated with increased CD3^+^ T lymphocyte infiltration into the tumors. Similarly, Shimoda and colleagues [20] found the prophylactic and therapeutic function of AAH-loaded DCs in a HCC-bearing BALB/c mice model. So far, inducible cellular immunity against AAH in murine models has found that AAH could be a promising immunotherapeutic target of HCC, but there are few reports regarding clinical trials of vaccine that employ this antigen; consequently, the efficiency of human's T cell responses specific for AAH is still unknown. In the study by Tomimaru et al. [21], synthesized HLA class I- and class II-restricted AAH peptides were found to be significantly immunogenic. In addition, both CD4^+^ and CD8^+^ T cells purified from the peripheral blood mononuclear cells of HCC patients were found to be activated by AAH protein-loaded DCs, and the inducement function of AAH protein-loaded DCs was remarkably stronger than that of AFP-loaded DCs [20]. To sum up, all the related researches suggest that AAH-loaded DC vaccine may represent as a novel candidate for the immunotherapy of HCC.

Constructing a stable delivering system carrying AAH gene is the first step to further explore the function of AAH-loaded DCs. In this study, we chose Ad as the optimal vector for the following reasons: (1) Ad possesses a strong ability of infection with respect to many different kinds of cells; (2) recombinant Ad could induce cellular immune response and/or humoral immune response; and (3) it infects host cells without insertion mutation, comparatively more safely [22, 23]. As a result, Ad is more likely to be chosen in clinical trials of gene therapy compared with other kinds of vectors. Here, we introduced a gateway recombinant cloning technology, a highly efficient and accurate cloning method, to construct a new-type recombinant Ad vector encoding AAH gene. Unlike conventional cloning technology, the gateway technology provides an accurate way on the basis of bacteriophage-*λ* to transfer DNA fragments between cloning vectors and allows precise cloning without altering the coding sequence in both donor and destination vectors because of the presence of *ccd*B gene [24, 25]. Furthermore, the gateway technology performs an effective cloning of objective DNA fragments in a single step, yielding destination vectors rapidly with the absence of restriction enzymes and ligases [26]. As a consequence, we highly appreciate the potential of gateway technology for constructing recombinant Ad vector. In this study, pAd CMV/V5-DEST, a 2nd-generation Ad vector, was selected as the destination vector. In the BP reaction, the attB-flanked PCR products were inserted into the donor vector containing the attP sites. Then, the entry clone with the attL sites and pAd CMV/V5-DEST with the attR sites reacted with each other to yield the final expression clone. The whole process was simple and convenient, required much less time, and greatly saved cost. The destination protein was found to have steadily expressed, as demonstrated by the positive expression of AAH protein in postinfected HEK293 cells after 13 days of infection. It was noteworthy that the ability of such recombinant Ad, stably expressing AAH protein, could make it be more beneficial to consistently produce antigens so as to effectively induce immune response in the body.

Ad has a broad spectrum of infection. Not only HEK293 cells but also DCs could be highly effectively infected by Ad in vitro. DCs infected by recombinant Ad vector harboring AAH gene were found to have matured because increased expressions of CD11c, CD80, and MHC-II on the surface of AAH-DCs were detected. It is well known that “mature” DCs have a more powerful ability of antigen presenting than “immature” DCs, which is achieved by a mechanism known as cross-presentation. In the present study, the expression of AAH protein in DCs was successfully detected after 24–72 hours of infection (data not supply), implying that DC vaccine carrying AAH antigen was successfully constructed. Proliferation and apoptosis are two important properties of tumor cells. The abilities of inhibiting proliferation and prompting apoptosis were often used to define whether the antitumor method is effective or not. The antitumor mechanism of DC-based vaccine is that DCs could induce both CD4^+^ T helper cells and CD8^+^ CTLs resulting in activating apoptosis signal pathways in tumor cells [27]. In the current study, we observed that AAH-DCs cocultured with T lymphocytes had a stronger cell-killing effect against HepG2 cells than the control groups in vitro. We also found that the AAH-DC vaccine could induce more effective cellular immune responses in vivo when compared with the control groups. Although our study was limited in HCC cell lines, the results supplied us with a theory and practice basis for exploring the antitumor function of CTL immune response in animal models. A report by Wu et al. [28] has shown that DCs loaded with Ad vector carrying human papillomavirus-16 E6/E7 (HPV-16 E6/E7) fusion gene cocultured with isogenous T cells induced CTL response resulting in a remarkably lethal effect on cervical cancer cell CaSki in vitro. Fu et al. [29] also reported the significant inhibition function of CTLs elicited by HPV E6/E7-loaded DCs against tumor-bearing nude mice. The above data support the hypothesis that Ad vector harboring antigen plays a role in constructing DC-mediated tumor vaccine.

## 5. Conclusions

In conclusion, the current study identified that the gateway recombinant cloning technology is a powerful and efficient method of constructing recombinant adenovirus. The destination product, Ad-AAH-IRES2-EGFP, combining with DCs could significantly kill HepG2 cells in vitro. We propose that the AAH-DC vaccine may be a potential candidate for immunotreatment of HCC and other types of malignancies expressing AAH.

## Figures and Tables

**Table 1:** PCR primer sequences of the AAH gene for DNA ligating.

Primer name	Sequence (5′→3′)	Amplification fragments
AAH-F	ATCATCCTCGAGGCCACCATGGCCCAGCGTAAGAATGCCAAG	2277 bp
AAH-R	ATCATCGGATCCCTAAATTGCTGGAAGGCTGCGTCTCTGCT	

**Table 2:** PCR primers for AAH and IRES2-EGFP fusion.

Primer name	Sequence (5′→3′)
attB1-AAH	GGGGACAAGTTTGTACAAAAAAGCAGGCTTCGCCACCATGGCCCAGCGTAAGAATGCCAAGAGC
IRES2-AAH-2277R	GTTAGGGGGGGGGGAGGGAGAGGGGCCTAAattGCTGGAAGGCTGCGTCTCTGCT
AAH-IRES2-1F	AGCAGAGACGCAGCCTTCCAGCAattTAGGCCCCTCTCCCTCCCCCCCCCCTAAC
attB2-EGFP	GGGGACCACTTTGTACAAGAAAGCTGGGTCTTACTTGTACAGCTCGTCCATGCCGAGAGTG

## Data Availability

The data used to support the findings of this study are available from the corresponding author upon request.
